# Construction of a SNP-based genetic linkage map in cultivated peanut based on large scale marker development using next-generation double-digest restriction-site-associated DNA sequencing (ddRADseq)

**DOI:** 10.1186/1471-2164-15-351

**Published:** 2014-05-09

**Authors:** Xiaojing Zhou, Youlin Xia, Xiaoping Ren, Yulin Chen, Li Huang, Shunmou Huang, Boshou Liao, Yong Lei, Liyin Yan, Huifang Jiang

**Affiliations:** Key Laboratory of Biology and Genetic Improvement of Oil Crops, Ministry of Agriculture, Oil Crops Research Institute of the Chinese Academy of Agricultural Sciences, Wuhan, 430062 Hubei People’s Republic of China; Nanchong Academy of Agricultural Sciences, Nanchong, 637000 Sichuan People’s Republic of China; Databridge Technologies Corporation, Wuhan, 430062 Hubei People’s Republic of China

**Keywords:** Cultivated peanut, Linkage map, SNP, ddRADseq

## Abstract

**Background:**

Cultivated peanut, or groundnut (*Arachis hypogaea* L.), is an important oilseed crop with an allotetraploid genome (AABB, 2*n* = 4*x* = 40). In recent years, many efforts have been made to construct linkage maps in cultivated peanut, but almost all of these maps were constructed using low-throughput molecular markers, and most show a low density, directly influencing the value of their applications. With advances in next-generation sequencing (NGS) technology, the construction of high-density genetic maps has become more achievable in a cost-effective and rapid manner. The objective of this study was to establish a high-density single nucleotide polymorphism (SNP)-based genetic map for cultivated peanut by analyzing next-generation double-digest restriction-site-associated DNA sequencing (ddRADseq) reads.

**Results:**

We constructed reduced representation libraries (RRLs) for two *A. hypogaea* lines and 166 of their recombinant inbred line (RIL) progenies using the ddRADseq technique. Approximately 175 gigabases of data containing 952,679,665 paired-end reads were obtained following Solexa sequencing. Mining this dataset, 53,257 SNPs were detected between the parents, of which 14,663 SNPs were also detected in the population, and 1,765 of the obtained polymorphic markers met the requirements for use in the construction of a genetic map. Among 50 randomly selected *in silico* SNPs, 47 were able to be successfully validated. One linkage map was constructed, which was comprised of 1,685 marker loci, including 1,621 SNPs and 64 simple sequence repeat (SSR) markers. The map displayed a distribution of the markers into 20 linkage groups (LGs A01–A10 and B01–B10), spanning a distance of 1,446.7 cM. The alignment of the LGs from this map was shown in comparison with a previously integrated consensus map from peanut.

**Conclusions:**

This study showed that the ddRAD library combined with NGS allowed the rapid discovery of a large number of SNPs in the cultivated peanut. The first high density SNP-based linkage map for *A. hypogaea* was generated that can serve as a reference map for cultivated Arachis species and will be useful in genetic mapping. Our results contribute to the available molecular marker resources and to the assembly of a reference genome sequence for the peanut.

**Electronic supplementary material:**

The online version of this article (doi:10.1186/1471-2164-15-351) contains supplementary material, which is available to authorized users.

## Background

Cultivated peanut, or groundnut (*Arachis hypogaea* L.), is a major economic crop in most tropical and subtropical areas of the world and represents a significant source of oil and protein for human nutrition. Because this species is a self-pollinating allotetraploid (AABB, 2n = 4× = 40) with a large genome size (2800 Mb/1C) and a narrow genetic base, leading to very low DNA polymorphism, the development of molecular markers and genomic resources in peanut has always been a formidable task [1–3]. In recent years, many efforts have been made to construct linkage maps as the genetic basis for quantitative trait locus (QTL) analyses of important, complex traits. However, almost all the maps constructed using low-throughput molecular markers, e.g., restriction fragment length polymorphisms (RFLPs) and simple sequence repeats (SSRs), present a low density and are unable to provide precise information on the QTLs controlling the traits of interest [4–6]. In the tetraploid peanut, almost all of the existing linkage maps for single populations include fewer than 350 markers [5, 7], with the exception of two recently developed linkage maps that include over 1000 markers [8, 9]. In 2012, a single nucleotide polymorphism (SNP) marker-based genetic map was successfully constructed for the AA genome due to the greater simplicity of diploids [10], marking a step forward in the development of SNP markers for peanut. However, until recently, only sporadic SNP markers had been developed in cultivated peanuts, and no SNP marker-based genetic map has been previously reported.

SNPs are widely distributed in the genome and are the most abundant type of DNA variation currently used as a genetic marker [11]. Compared to markers based on size discrimination or hybridization, SNPs directly interrogate sequence variation and can reduce genotyping errors [12]. SNP discovery is amenable to high-throughput technology, such as next-generation sequencing (NGS) technologies, which produce DNA sequences at a rate several orders of magnitude faster than conventional methods, making them an excellent tool for use in genomics research.

The complexity of genomes can be overcome by using reduced representation libraries (RRLs), and the combination of RRLs with multiplex sequencing can improve the throughput of SNP identification and genotyping [13, 14]. RRLs are being used in a wide range of applications, including the construction of linkage maps, fine mapping of genes and association studies [15–17]. RRL was first and has usually been demonstrated through restriction site-associated DNA (RAD) tagging and NGS of RAD tags [18, 19]. To increase the breadth of RADseq applications, the double-digest restriction-site-associated DNA sequencing (ddRADseq) method was developed by eliminating random shearing and explicitly using size selection to recover a tunable number of regions [20]. ddRADseq tags not only possess the advantages of RAD tags, such as allowing high-throughput, multiplexed sequencing and being amenable to genotyping, but they also provide improved efficiency and robustness compared to RAD. In *Brassica napus*, RRLs were constructed for two parents and 91 of their doubled haploid (DH) progenies using the ddRADseq technique, and restriction fragments in the size range of 141–420 bp were chosen to represent the reduced genome and to allow multiplex sequencing to be conducted [21]. SNPs were identified and genotyped from the high-quality polymorphism data, and a SNP bin map comprising 8,780 SNP loci, together with 47 SSR loci was constructed. Recknagel et al. [22] applied this technology to obtain a high-density linkage map for Cichlid fishes. A total of 755 markers were genotyped in 343 F_2_ hybrids. The map resolved 25 linkage groups and spanned a total distance of 1,427 cM, with an average marker spacing distance of 1.95 cM [22]. These data suggest that ddRADseq technology can contribute to the construction of linkage maps through the identification and genotyping of SNPs across large numbers of individuals for a range of markers in both model and non-model species.

Through the utilization of NGS data, several bioinformatics approaches and tools have been developed for SNP discovery and genotyping in complex genomes. For instance, the GMAP alignment method and the Maq analysis method have been applied in soybean with stringent matching criteria (using only high-quality reads, unique mappings, multiple-reads SNP support) for high-throughput SNP discovery through RRL resequencing. Both of these methods predicted large numbers of SNPs, and the validation rate ranged from 79% to 92.5% [23]. The Universal Network-Enabled Analysis Kit (UNEAK) approach was developed for SNP discovery in switchgrass, which is a highly heterozygous polyploid (tetraploid and octoploid) species lacking a reference genome, and a total of 1.2 million putative SNPs were discovered in a diverse collection of primarily upland, northern-adapted populations [24]. In a study on hexaploid cultivated oat plants, contigs were filtered through a bioinformatics pipeline to eliminate ambiguous polymorphisms caused by subgenome homology. This procedure identified 9,448 candidate SNP loci. The greatest attrition of these candidate SNPs was based on SNP conservation between reads from a single germplasm, and 55% *in silico* SNPs were rejected [12].

Genetic linkage maps based on molecular markers can form the basis for QTL mapping and marker assistant selection and permit the elucidation of genome structure and organization. For instance, in the Tifrunner × GT-C20 cultivated peanut population, using the F_2_ and F_5_ generations, Wang et al. [7] and Qin et al. [25] constructed two genetic maps with 318 and 239 loci, respectively. Both genetic maps were compared to the reference consensus genetic map that was developed by Gautami et al. [26] for anchor and colinearity analysis [27]. Using the two maps and the combined multi-environment phenotyping data, Wang et al. [27] identified QTLs for thrips, tomato spotted wilt virus (TSWV), and leaf spot (LS). Although available linked markers of important traits are still lacking in peanut, we are hopeful about the future of marker-assisted breeding from its successful application of converting the peanut cultivar Tifguard [28] into ‘high oleic Tifguard’ in 28 mo [29] using the available limited resources in peanut.

In this study, we employed the ddRADseq approach to achieve mass discovery of SNP markers for cultivated peanut. A bioinformatics pipeline was applied for SNP calling in the parents and genotyping in the progeny. Using the newly developed markers and previously published SSR markers, a SNP-based genetic map was constructed. The characterization of this genetic map and the comparative analysis with a previous integrated consensus map were performed.

## Results

### Library construction and sequencing results

The ddRADseq protocol was used to construct reduced-representation libraries for the parents Zhonghua 5 and ICGV86699 and 166 of their RIL progenies. A rare-cutting restriction enzyme, SacI (GAGCTC), and a restriction enzyme with a more common recognition site, MseI (TTAA), were chosen based on previous success in reducing genome complexity [21]. The selected size of the DNA fragments for the ddRADseq library was 300 bp to 500 bp (with indices and adaptors). To enable multiplex sequencing of the libraries, we used a set of molecular identifying sequences (MIDs) ranging in length from 5 bp to 8 bp that allowed reads to be assigned to unique individuals. Each sequence contained adaptors, which included the sequencing primer, MID and complimentary sequence to the overhangs produced by the restriction enzymes,followed by locus-specific genomic DNA. Libraries from 12 different individuals tagged with 12 barcodes were pooled and sequenced on the Illumina HiSeq2000 platform.

**Figure 1:**
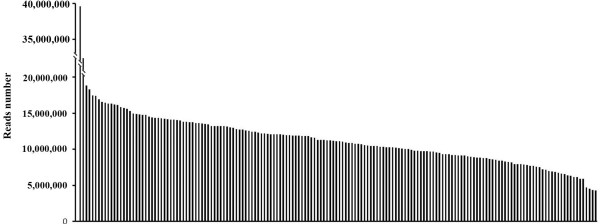
**The numbers of sequencing reads for the RIL individuals and their parents.** The first two bars of the x-axis indicate Zhonghua 5 and ICGV86699, and the following bars represent the 166 RIL lines generated from these parents; the y-axis indicates sequencing reads.

Massively parallel Solexa sequencing of the ddRADseq library generated ~175 Gb of data containing 952,679,665 paired-end reads, with each read being ~90 bp in length. The Q20 (representing a quality score of 20, indicating a 1% chance of error and, thus, 99% confidence) ratio was 96.7%, and the guanine-cytosine (GC) content was 44.3%. Among these high-quality data, 72 million reads came from the parents (39,589,594 reads from Zhonghua 5 and 32,410,406 reads from ICGV86699), and ~ 1,833 million reads came from the libraries for the 166 F_9_ progeny. In the RILs, the number of reads per F_9_ individual ranged from 3,940,624 to18,828,436, with an average of 11,044,333 reads (Figure 1).

### SNP calling between the parents

The sequencing reads of the parents were clustered using Vmatch software [30]. The number of reads forming each cluster showed eight-fold average sequencing coverage. The consensus sequences contained a total of 71,590,118 sequence tags, and the total length of the consensus sequences was 214,422,448 bp. SNP calling between the parents was performed by aligning the reads from the parents to the consensus sequences using SOAP software [31]. A total of 39,357,846 (99.4%) reads from Zhonghua 5 and 32,232,272 (99.5%) reads from ICGV86699 could be aligned to the consensus sequences. We chose uniquely mapped reads for SNP discovery. The sequences that matched more than 50 locations in the consensus sequences corresponded to 20,567 events and represented serious contaminating repetitive elements. In this case, a total of 30,977,293 (43%) reads were rejected because of multiple matching loci. Of the 40,612,825 remaining unique reads, 1,346,253 loci were eliminated because of heterozygous alleles within one parent, and 31,010 loci were removed due to less than four reads being found in each line. After applying the filtering procedure, 53,257 SNP loci between the parents were retained.

### SNP genotyping of the RIL population

**Figure 2:**
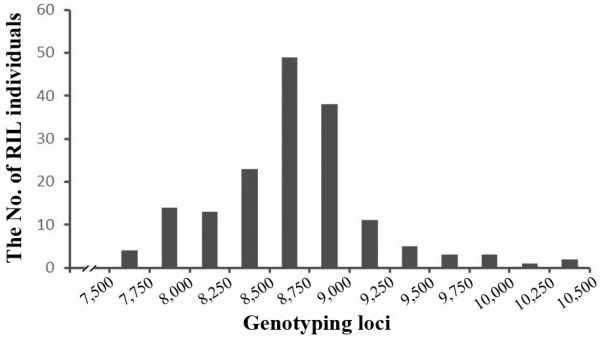
**Distribution of genotyping loci for RIL individuals.** The x-axis indicates the number of genotyping loci; the y-axis indicates the number of RIL individuals.

Because the construction of a SNP-based genetic map required the polymorphic markers between the two parents, the consensus sequences that did not contain SNPs were discarded, producing a reduced consensus sequences of 7,422,496 bp. Calling of SNP genotypes was performed in the population based on aligning the sequencing reads for the RIL lines to the reduced consensus sequences. A total of 516,699,812 sequencing reads from RIL individuals were aligned to the reduced consensus sequences, and the average number of aligned reads per individual was 3,112,649. Among the total aligned reads from the RIL individuals, 191,321,469 were for unique sites, and the average number of uniquely mapped reads per individual was 1,152,539, accounting for 37% of the average aligned reads for individuals. The uniquely mapped reads were chosen for subsequent SNP discovery. A total of 609,578 SNP loci were removed based on showing a heterozygous genotype, and 10,032 loci were removed due to an insufficient read depth (≤4). We detected 14,663 SNPs in the RIL population. For each individual from the RILs, the number of genotyping loci ranged from 7,606 to 10,429, averaging 8,646, and the majority of individuals presented 7750–9250 genotyping loci (Figure 2). Using a maximum missing data (MMD) threshold of 25% in the RIL population for each locus, a total of 1,765 SNP loci were finally recovered. The SNP-flanking sequences and the polymorphic sites are listed in Additional file 1.

### SNP analysis and validation

**Figure 3:**
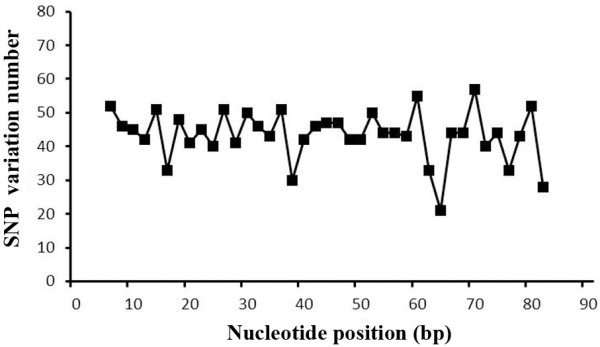
**The distribution of the total number of SNP variations at each nucleotide position for each read.**

In total, the stringent *in silico* SNP selection criteria produced 1,765 SNPs, and the SNP distribution and the percentages of different SNP types were investigated. The SNPs were distributed evenly across the reads, with the end showing a slightly broadening range, mainly due to the decline of the base quality (Figure 3). Most of the SNPs were transition-type SNPs, with the C/T and G/A types accounting for 37% and 36% of the SNPs, respectively. The other four SNP types were transversions, which included C/G, G/T, C/A, and A/T, showing percentages ranging from 3% to 11%, accounting for 27% of all SNPs (Table 1).

**Table 1:** **Statistics for the identified SNP marker types**

Type of variation	Number	Proportion of type
C/G	55	3%
G/T	144	8%
C/A	186	11%
A/T	91	5%
C/T	652	37%
G/A	637	36%
Total	1765	100%

To investigate the authenticity of the identified SNPs, we randomly selected 50 SNPs for validation of single nucleotide variations. PCR primers were designed to amplify the fragments containing the SNPs. We further sequenced the PCR products for all 50 loci amplified from the two parents using the Sanger sequencing method to confirm these SNPs. Of these 50 SNPs, 47 (94%) could be confirmed by Sanger sequencing. All 47 confirmed SNPs showed the expected nucleotide variations, while among the remaining 3 SNPs, 1 failed to amplify clearly by PCR, and 2 were a mixture of the expected allelic variations and homoeologous sequences. These results further demonstrated the efficacy of this approach for discriminating allelic SNPs from cultivated peanut.

### A. hypogaea genetic map

**Figure 4:**
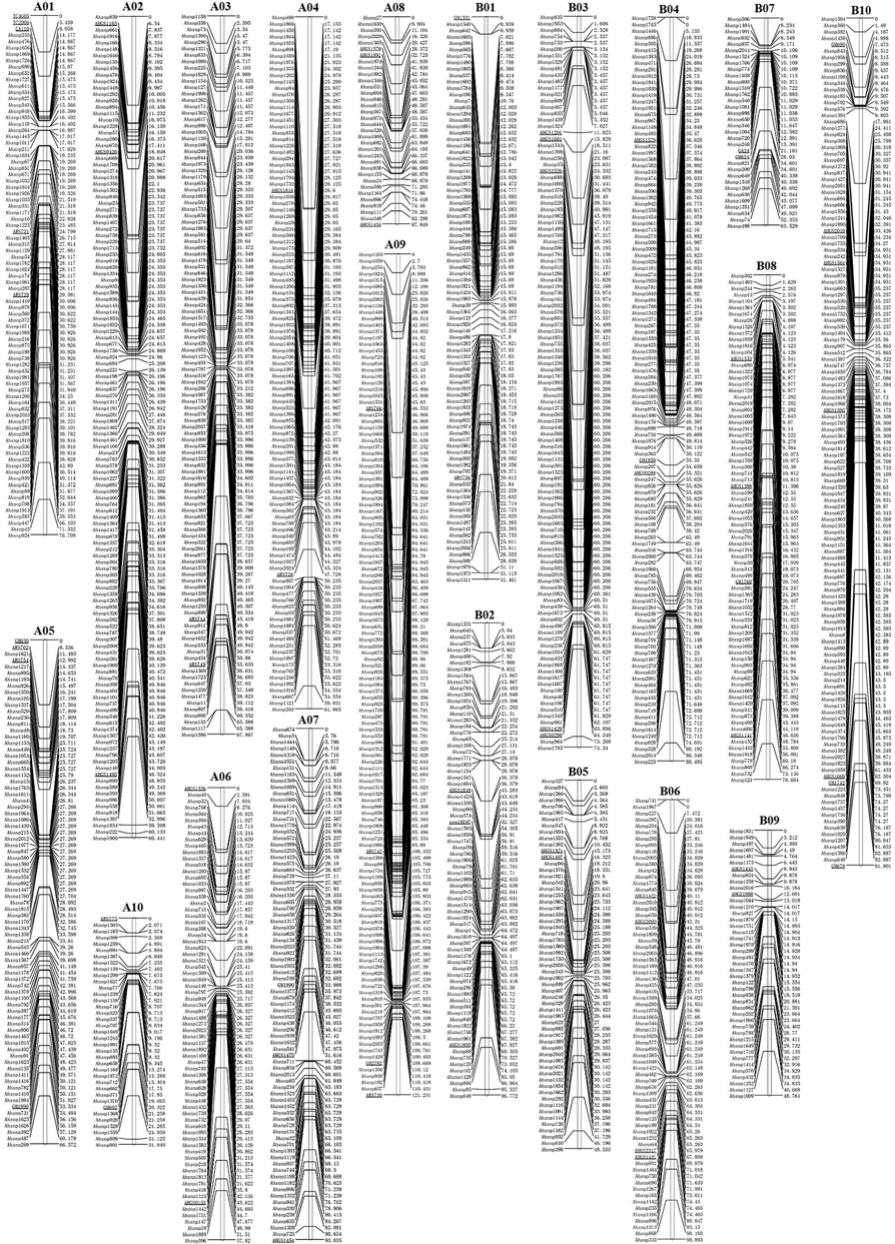
**The SNP-based genetic linkage map for cultivated peanut using the Zhonghua 5 × ICGV86699 population.** SNP markers are preceded by ‘Ahsnp’. Markers are shown on right side of the LGs, while map distances are shown on the left side. Sixty-four previously published markers (underlined) were selected from the integrated consensus map of Shirasawa et al. (2013) or Gautami et al. (2012) to assign the linkage map to the corresponding chromosome.

Of the 1,765 developed SNP markers, 1,621 were included in the *A. hypogaea* map, which were combined into 20 linkage groups (Figure 4). To anchor and align the current map with previously published maps for peanut, 379 previously published SSR markers for single loci distributed among the 20 linkage groups of the integrated consensus map, which came from Shirasawa et al. [9] or Gautami et al. (2012) [26], were screened on the parental genotypes. As a result, 103 polymorphic markers were identified. A total of 64 markers were mapped to the 20 LGs of the current map (Table 2; Additional file 2).

**Table 2:** **Characteristics of the molecular markers used for mapping**

Molecular markers	Number of polymorphism primers	Number of linked markers	Number of unlinked markers	Frequency of unlinked markers (%)
SNP	1765	1621	144	8.2%
SSR	103	64	39	37.9%
Total	1926	1685	241	23.1%

Overall, the linkage map contained 1685 loci, and covered a total of 1446.7 cM, forming 1267 bins. The LGs ranged from 31.5 to 121.2 cM in length, and seven linkage groups contained over 100 marker loci. B07 and A08 were the smallest LGs, spanning 63.5 cM and 87.8 cM, respectively, and comprising 34 loci, whereas A09 was the largest LG, spanning 121.2 cM and containing 132 loci. The marker densities ranged from 0.4 cM/locus in B01 to 2.7 cM/locus in A08, resulting in an average distance of 0.9 cM between markers for the entire map (Table 3).

**Table 3:** **Features of the 20 linkage groups**

LGs	Length (cM)	No. of loci	No. of bins	Density (cM/locus)	No. of Distorted loci	No. of SDRs^a^	No. of the longest SDRs	Frequency of segregation distortion marker	Largest gap (cM)	Gaps ≤ 5
A01	76.8	83 (5)	55	0.9	25	2	12	30.1%	6.6	93.9%
A02	60.4	129 (3)	102	0.5	5	1	3	3.9%	6.5	98.4%
A03	67.9	113 (2)	73	0.6	44	4	16	38.9%	5.0	99.1%
A04	62.0	109 (2)	72	0.6	24	1	22	22.0%	17.1	98.1%
A05	66.6	80 (4)	62	0.8	43	3	25	53.8%	8.5	94.9%
A06	57.8	72 (2)	53	0.8	5	1	5	6.9%	6.3	95.8%
A07	93.0	81 (3)	71	1.2	16	3	4	19.8%	8.8	93.8%
A08	87.8	34 (3)	31	2.7	6	0	0	17.7%	9.9	84.8%
A09	121.2	132 (4)	111	0.9	14	2	4	10.6%	13.6	93.9%
A10	31.8	37 (2)	31	0.9	14	2	3	37.8%	6.2	97.2%
B01	31.5	89 (2)	67	0.4	65	8	9	73.0%	6.1	98.9%
B02	96.8	75 (3)	59	1.3	45	2	40	60.0%	10.9	90.5%
B03	74.3	115 (5)	56	0.7	28	1	22	24.4%	8.9	98.2%
B04	88.5	117 (3)	97	0.8	64	3	52	54.7%	7.7	94.8%
B05	48.5	59 (2)	47	0.8	32	4	15	54.2%	4.7	100%
B06	98.9	70 (4)	55	1.4	9	1	5	12.9%	12.9	89.9%
B07	63.5	34 (2)	29	1.9	21	2	9	61.8%	11.2	81.8%
B08	78.7	80 (4)	61	1.0	50	2	47	62.5%	6.9	94.9%
B09	48.8	43 (2)	38	1.2	36	2	26	83.7%	8.1	95.2%
B10	91.9	133 (7)	97	0.7	113	3	58	85.0%	11.6	96.2%
Total	1446.7	1685 (64)	1267	0.9	659	47	/	/	/	/
Mean	72.3	84	63	/	/	/	/	/	/	94.5%

The number in parentheses is represents the number of SSR anchor loci in the linkage group.

^a^SDRs, segregation distortion regions.

‘Gap ≤ 5’ indicates the percentages of gaps in which the distance between adjacent markers was smaller than 5 cM.

**Figure 5:**
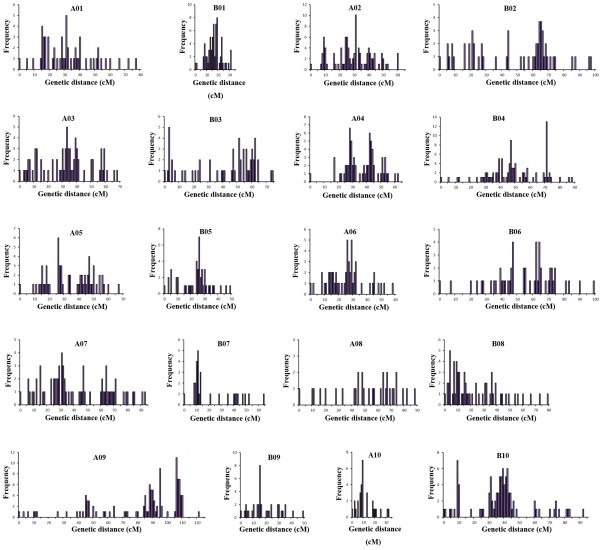
**The X axis indicates the position in each linkage group in 1 cM intervals, and the Y axis indicates the number of bins within 1 cM.**

In the map, 659 (39.1%) markers showed a skewed segregation pattern (*P* < 0.05; Table 3). The segregation distortion markers were distributed among every LG. The average number and proportion of distorted markers of the LGs in the A sub-genome were 196 and 22.5%, respectively, which were lower than in the B sub-genome (463 and 56.8%, respectively; Table 3), suggesting that the chromosomal selection in the A sub-genome has smaller scale than that in the B sub-genome. The majority of the distorted markers were distributed as clusters, and 47 segregation distortion regions (SDRs) were detected and distributed in all of the linkage groups except A08. B01 had the largest number of SDRs, and B10 contained the longest SDR, which included 58 markers, spanning a distance of 24.3 cM. The degree of linkage between markers was reflected by the fact that ‘Gap **≤** 5’ was observed with an average value of 94.5%. A total of 7 regions of the linkage groups contained gaps of more than 10 cM, and the largest gap in this map was 17.1 cM, located in A04 (Table 3, Figure 5).

### Comparative analysis

The linkage map in this study was aligned to the integrated consensus map developed by Shirasawa et al. [9]. The main marker types in this integrated consensus map were SSRs and transposons. In 64 single-locus SSR loci of the SNP-based linkage map, 56 were identified as having corresponding loci in the 20 chromosomes of the integrated consensus map (Additional file 3), while the remaining SSR markers were from another integrated map developed by Gautami et al. [26]. The aligned single-locus SSR loci of this SNP-based map could be treated as anchors to assign linkage groups (LGs) to specific chromosomes. Although a direct alignment of SNPs with SSRs or transposon markers is not practical, an indirect alignment of the different marker types through the GSSs sequences of peanut from NCBI is feasible. The different types of markers that map to the same sequence fragments were considered as having similar or adjacent map positions. From the alignment, 90 loci distributed on 20 linkage groups of the newly developed linkage map were identified as having corresponding loci in the integrated consensus map (Additional file 3). The corresponding LGs were collinear, except LG B03. Within the conserved regions, the orders of some of the conserved loci were altered by simple inversions or translocations. For collinear LGs, such as LG A03, two SSR markers and seven SNP markers could be mapped in the integrated consensus map, giving conserved consistent points of corresponding LG. For LG B03, 7 corresponding markers were clustered into two chromosomal segments. The first of these was roughly collinear, with 4 aligned markers spanning 18.4 cM (24.8%) on the SNP-based map and 35.5 cM (24.5%) on the integrated consensus map. The other fragment had a reversed order with 3 aligned markers spanning 12.5 cM (16.8%) on this map and 23.2 cM (16.0%) on the integrated consensus map. This observation was similar as the comparative analysis between the integrated consensus map and the TF6 population [9].

## Discussion

*A. hypogaea* is a recently formed tetraploid that most likely originated from natural hybridization of the mesopolyploids *A. duranensis* and *A. ipaensis*, which contributed to the constituent A and B genomes, respectively. In recent years, many studies of SNP development in polyploid crops have been reported. Trick et al. (2009) [32] exploited a methodology including computational tools and detected 36,424 (87.5%) hemi-SNPs and 5,169 (12.4%) simple SNPs between two rapeseed cultivars under a requirement for a minimum of four reads depth using Solexa transcriptome sequencing. Based on this study, Hu et al. (2012a, 2012b) [33, 34] developed a new method for identifying SNP markers in *Brassica napus* with filtering criteria based on the incorporation of read redundancy, quality index and lines information. Among these criteria, choosing only the unique sequences that match exactly one position in the reference genomes for SNP discovery is a particularly important filtering criterion and can greatly decrease the disturbance of paralogs within two lines. Hu et al. 2012a [33] identified 60,396 ‘simple SNPs’, and two associated SNPs were finally mapped to a major QTL region. Hu et al. 2012b [34] detected 655 SNPs, and the validation rate reached 84%. In the present study, to decrease complexity and improve the accuracy of genotyping, we developed markers using read mapping uniqueness as a filtering criterion, too. Combined with other filtering criteria, the SNP sites were considered to be simple SNPs if there were no less than four reads depth for each genotype that revealed the same base change. In total, 53,720 SNPs were identified between the two parents, and 1,765 polymorphic markers were identified for genetic linkage map construction. Forty-seven out of 50 SNPs (94%) were verified according to Sanger sequencing, showing that the applied bioinformatics analyses were stringent and effective.

In the current study, a linkage map was finally constructed that was comprised of 1,685 marker loci, including 1,621 SNPs and 64 SSR loci, and spanning 1446.7 cM. The map was divided into 20 linkage groups and assigned to corresponding chromosomes. The first linkage map anchored to the A and B genomes was published by Foncéka et al. [5] and included 298 loci in 21 linkage groups (LGs) from a cultivated BC_1_F_1_ population. Because of the low marker density in the existing population-specific linkage maps and the difficulty of understanding the genomic structure of Arachis, two significant integrated consensus maps were recently constructed based on the segregation genotypes of multiple populations, anchored to 20 consensus LGs corresponding to the A and B genomes (A01-A10, B01-B10) [9, 26]. In this study, the applied SSR markers amplified single loci, distributed among the 20 linkage groups from the above two integrated consensus maps. The subsequent linkage analysis generated a total of 20 linkage groups. The present linkage map corresponds to the number of chromosomes (n = 20) in cultivated peanut, and the linkage groups were assigned to the specific chromosomes.

Segregation distortion is a common biological phenomenon and is one of the engines driving evolutionary processes. It can be observed in almost all types of hybrid segregating populations. In general, the skewed segregation ratio of RIL populations is higher than that of backcross populations (BC) and doubled haploid populations (DH). F_2_ populations show the lowest skewed segregation ratio [35]. The genetic basis of segregation distortion is still under debate, and gametophyte and/or zygotic selection and chromosomal rearrangements may be the main cause of this phenomenon. Studies have demonstrated that a large number of segregation distortions and SDRs occur in many species, such as maize [36], barley [37], and potato [38]. In this study, we used a RIL F_9_ population as a mapping population to construct a linkage map, and 659 (39.1%) markers showed skewed segregation. This high-generation population could improve the accuracy of bioinformatics analysis for SNP discovery because of long stretches of consecutive homozygous genotypes, while the marker more likely skewed segregation probably related to the many generations of natural selection and artificial sampling involved in the construction of the RIL population. In this map, most of the markers exhibiting segregation distortion were distributed as clusters in linkage groups. Distorted markers were often strung together, suggesting that there has been selection for gametophytes or sporophytes.

As discussed above, both the SNP and SSR markers on this map presented single-locus nature in the AABB genome. Comparative analysis between the AA and between BB genomes were performed and showed that all LGs in the SNP-based map were collinear with their corresponding LGs in the integrated consensus map, except LG B03, for which the corresponding markers were clustered into two chromosomal segments and had reversed orders. This observation suggested the chromosome segment with inversion or rearrangement in LG B03. Relative to the large peanut genome, the number of markers is still low and the available peanut sequence is limited and the common GSSs that can be used as bridges to align SNP and SSR markers less. The completion of peanut genome sequence and the development of increasing numbers of molecular markers will establish more alignment points between the genetic maps with different types of markers. Even so, the alignment of some parts of the present map with integrated consensus maps of peanut demonstrates the possibility of developing SNP markers for constructing linkage groups in cultivated peanut and improving our understanding of the genome.

The current version of the cultivated peanut linkage map is a considerable improvement compared with the previously available versions (Table 4). There are two major reasons for this improvement. First, this is the only SNP-based linkage map that has been produced for cultivated peanut. Initially, genetic maps were developed for wild species with AA- and BB- genomes. For cultivated peanut species or crosses of cultivated and synthetic tetraploid peanut species, a few linkage maps have recently been constructed (Table 4), and some of these maps were based on multiple populations. Earlier maps used RFLP or AFLP markers, while the later maps were mainly based on SSR markers. Varshney et al. [39] constructed the first SSR linkage map for cultivated peanut. Since that time, the construction of SSR-based genetic linkage maps for *A. hypogaea* has proceeded rapidly. This study was the first to develop SNP markers on a large scale to construct a genetic map for cultivated peanut. Another obvious improvement is that the maximum number of markers for a linkage map in a single mapping population was used. Shirasawa et al. [8] published a high-density genetic map composed of 1,114 loci, including SSR and transposon markers. Another high-density map included 1,469 loci, with an average distance of 1.0 cM between adjacent loci [9]. The map produced in the present study contains 1,685 markers, and the average genetic interval is 0.9 cM per marker. To our knowledge, the number of markers included in this map is the highest among the available population-specific linkage maps for tetraploid peanuts (Table 4).

**Table 4:** **Comparison of tetraploid linkage maps for Arachis from a single population**

Cross combination	Population type	Types of markers	Number of markers	Maps length(cM)	Groups	References
*A. hypogaea* 'Florunner' × (*A. batizocoi* 'K9484' × (*A. cardenasii* 'GKP10017' × *A. diogoi*' GKP10602') ^4×^)	BC_1_F_1_	RFLP	370	2210	23	[4]
*A. hypogaea* 'ICG12991' × *A. hypogaea* 'ICGV-SM 93541'	F_2_	AFLP	12	139.4	5	[40]
*A. hypogaea* 'TAG24' × *A. hypogaea* 'ICGV 86031'	RIL	SSR	135	1270.5	22	[39]
*A. hypogaea* 'Fleur11' × (*A. ipaënsis* 'KG30076' × *A. duranensis* 'V14167')^4×^	BC_1_F_1_	SSR	298	1843.7	21	[5]
*A. hypogaea* 'Yueyou 13' × *A. hypogaea* 'Zhen Zhuhei'	RIL	SSR	132	684.9	19	[41]
*A. hypogaea* 'Yueyou 13' × *A. hypogaea* 'FU 95-5'	RIL	SSR	109	540.69	21	[41]
*A. hypogaea* 'Yueyou 13' × *A. hypogaea* 'J 11'	RIL	SSR	46	401.7	13	[41]
*A. hypogaea* 'TAG 24' × *A. hypogaea* 'GPBD 4'	RIL	SSR	188	1,922.4	20	[6]
*A. hypogaea* 'TG 26' × *A. hypogaea* 'GPBD 4'	RIL	SSR	181	1,963	21	[6]
*A. hypogaea* 'ICGS76' × *A. hypogaea* 'CSMG 84-1'	RIL	SSR	119	2,208.2	20	[42]
*A. hypogaea* 'ICGS 44' × *A. hypogaea* 'ICGS 76'	RIL	SSR	82	831.4	15	[42]
*A. hypogaea* 'SunOleic 97R' × *A. hypogaea* 'NC94022'	RIL	SSR, CAPs	172	920.7	22	[25]
*A. hypogaea* 'Tifrunner' × *A. hypogaea* 'GT-C20'	F_2_	SSR	318	1674.4	21	[7]
*A. hypogaea* 'YI-0311' × *A. hypogaea* 'Nakateyutaka'	F_2_	SSR, transposon, SNP	326	1332.9	19	[8]
*A. hypogaea* 'Satonoka' × *A. hypogaea* 'Kintoki'	F_2_	SSR, transposon	1114	2166.4	21	[8]
*A. hypogaea* 'Runner IAC 886' × c*A. ipaënsis* 'K30076' × *A. duranensis* 'V14167')^4×^	RIL	SSR, transposon	1469	1442	20	[9]
*A. hypogaea* 'Zhonghua 5' × *A. hypogaea* 'ICGV86699'	RIL	SNP, SSR	1685	1441.1	20	This study

Molecular markers and genetic linkage maps are the pre-requisites for undertaking genetic mapping of important traits and molecular breeding activities in crops. The female parent of the RIL population Zhonghua 5 is a popular high-yield cultivar in China, but it is susceptible to late leaf spot. However, the male parent, ICGV86699, has excellent resistance to this disease (Additional file 4), which is the most widely distributed peanut disease in China. The tools generated in this study will accelerate the genetic research and the process of introgression of beneficial traits into preferred varieties of cultivated peanut, such as resistance to late leaf spot. Because the high-density linkage groups were constructed based on molecular markers developed at the whole-genome level, it will also serve as a reference for positioning sequence scaffolds on the physical map to assist in the assembly of the peanut genome sequence.

## Conclusions

In this study, we constructed RRLs for two parents and 166 of their RIL progenies using the ddRADseq technique. Combined with a next-generation sequencing approach, we detected SNPs in cultivated peanut through the adoption of appropriate filtering criteria and constructed a genetic map containing 1,621 SNP loci and 64 SSR loci distributed among 20 LGs. All LGs in the SNP-based map were collinear with their corresponding LGs in the integrated map, except B03, where chromosome segment inversions or rearrangements maybe involved. The results of this study will provide a useful resource for molecular markers, QTL mapping, molecular breeding, and facilitating the assembly of a reference genome sequence for the peanut.

## Methods

### Plant material

A RIL population including 166 F_9_ lines was developed from a cross between Zhonghua 5 and ICGV86699. The parent Zhonghua 5 is an early maturing, high-yield popular cultivar but susceptible to late leaf spot disease. The parent ICGV86699 is a breeding variety from strains of distant hybridization, and it has resistance to late leaf spot that was introgressed to *A. hypogaea* from *A. duranensis*. The population was developed in the experimental field of the Oil Crops Research Institute of the Chinese Academy of Agricultural Sciences in Wuhan, Hubei Province. Genomic DNA was extracted from young leaf tissue essentially as described by Grattapaglia and Sederoff (1994) [43].

### ddRADseq library construction and sequencing

The procedure was performed as described by Chen et al. (2013) [21] with some modifications. First, genomic DNA was double digested separately with restriction enzymes. The double digest reactions were carried out in a volume of 25 μl containing approximately 150 ng of genomic DNA, 5 U of SacI and MseI (Fermentas), and 1× buffer. The reaction mixture was incubated at 37°C for 6 hr and 65°C for 90 min. Second, the fragments were ligated with adaptors. The ligation reaction was conducted in a reaction volume of 50 μl at 16°C overnight, containing 10 pmol of SacI and MseI adaptors, and 1,000 U of T4 DNA Ligase (New England Biolabs [NEB]). To ensure that the digestion was complete, the digestions were performed again with the same enzymes. Each sample was then amplified via PCR in a 50 μl reaction volume, containing 50–100 ng of adaptor-ligated DNA fragments as a template, 1× HF buffer, 3.5 mM MgCl_2_, 0.4 mM dNTPs, 0.5 U of iProof polymerase (Bio-Rad), and 4 pmol of two overhang primers. PCR amplification was performed according to the following program: 98°C for 2 min, followed by 13 cycles at 98°C for 30 s, 60°C for 30 s, and 72°C for 15 s, and a final extension at 72°C for 5 min. The PCR products were run on a 2% agarose gel, and fragments of 300–500 bp were recovered from the gel. The samples from 12 individuals were pooled together, and DNA was isolated using a Gel Extraction Kit (Qiagen). The libraries were quantified using Qubit fluorometer (Invitrogen), Agilent 2100 (Agilent Technologies) and real-time quantitative PCR, then submitted for sequencing on the Illumina HiSeq2000 platform.

### In silico SNP identification and genotyping

**Figure 6:**
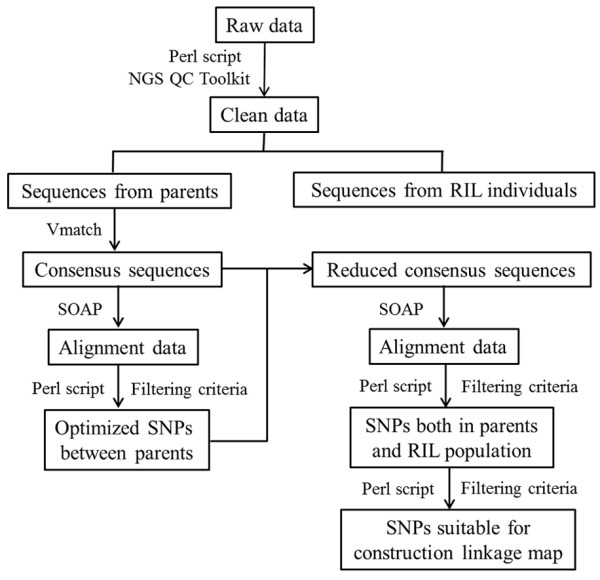
**Bioinformatics pipeline for SNP discovery.**

The bioinformatics process used for the identification of SNP markers is presented in Figure 6. Based on the Illumina raw data, a custom Perl script was written to sort sequences from individual samples based on indexes and trimmed barcode sequences for faster processing. Only sequences that presented an exact match to a barcode, followed by the expected sequence of nucleotides remaining after a SacI or MseI cut site were retained. The low-quality, contaminant sequences were trimmed using NGS QC Toolkit [44].

The cleaned data were clustered with Vmatch at a stringent level, where the default parameter setting was used, as applied in a number of SNP mining programs [30, 45]. Calling of single nucleotide polymorphisms (SNPs) was based on the alignment of the parental sequences to the consensus sequences using SOAP software [31]. Then, Custom Perl scripts were used for SNP calling according to published reports [46, 47]. The SNP calling fulfilled the following criteria: 1) to exclude regions of complex polymorphism, all PE reads from each line were aligned to the consensus sequences with at most two nucleotide mismatches on each strand of a read; 2) to avoid paralogue interference, only uniquely aligned reads were selected; 3) to avoid non-uniform polymorphisms, nucleotide variations present a 100% frequency within a genotype; and 4) to assure accuracy, every allele had to present a sequencing depth of no less than four reads. After identifying SNPs between parents, the SNP-containing sequences were extracted from the consensus sequences, thus producing reduced consensus sequences. For SNP detection in the RIL population, the same filtering criteria were used as in the parents. We calculated the likelihood of each line’s genotype using SOAPsnp [31]. A Bayesian model was applied, and the genotype with the highest probability was selected as the genotype of the individual at the specific locus. Each marker was required to have an allele present in at least 75% of F_9_ individuals, and each allele had to be present in at least 30 F_9_ individuals. Marker genotypes not meeting the minimum thresholds were scored as missing data.

### SNP validation through resequencing

Primer3plus was used to design primers to amplify the target fragments including the SNP variations. The SNPs that were validated between the two parents were subjected to genotype analysis in the RIL population. PCR amplifications were carried out in a volume of 20 μl, containing 100 ng of DNA template, 1 × Pfu buffer, 4 mM MgCl_2_, 0.4 mM dNTPs, 5 pmol of each primer, and 0.4 U of Pfu. Thermocycling was performed at 94°C for 3 min, followed by 35 cycles of 94°C for 30 s, 60°C for 1 min and 72°C for 45 s, with a final extension step of 72°C for 5 min, and then holding at 4°C. Aliquots (5 μl) of the PCR products were first analyzed on agarose gels to verify successful amplification, and the remaining PCR products were directly sequenced by BGI using an ABI3730 sequencer.

### Genetic linkage map construction

The RIL F_9_ population, consisting of 166 individuals, was utilized to construct a genetic map. The SNP marker sequences that were used for the genetic map are listed in Additional file 1: Table S1. The input datasets were constructed from 1,765 genotyped SNP markers and 103 previously published SSR loci. The program Joinmap 4.0 [48] was used to calculate the marker order and genetic distance. Recombination frequencies ≤ 0.45 and LOD scores ≥ 2.0 were used to create groups. The Kosambi mapping function was employed for map length estimations. Markers were tested for segregation distortion by the chi-square test. A graphic representation of the map was generated using Mapchart 2.0 software [49].

### Availability of supporting data

The Illumina sequencing data from this study have been deposited in the NCBI Sequence Read Archive under accession SRR1236437 (parents) and accession SRR1236438 (individuals of RIL population). The consensus sequences in this study have been deposited in LabArchives with doi: 10.6070/H45B00CC (https://mynotebook.labarchives.com/doi/NDgyMTQuNHwzNzA4OC8zNzA4OC9Ob3RlYm9vay8yNzQzMjEzNzI2fDEyMjM5MC40/10.6070/H45B00CC).

## Electronic supplementary material

Additional file 1: Table S1: Developed SNPs. The 1,765 SNPs and their flanking sequences. (XLSX 114 KB)

Additional file 2: Table S2: Details of previously published SSR markers. (XLSX 15 KB)

Additional file 3: Figure S1: Comparison between the LGs of the SNP-based map and the integrated consensus map. For each pair of aligned LGs, the left LG corresponds to the SNP-based map, and the right LG corresponds to the integrated consensus map. Horizontal lines on the LGs indicate the positions of the mapped loci. The loci of the common SSR markers and the SNP and SSR markers that have similar map positions between the corresponding LGs of the two maps are connected by black lines. (PDF 8 MB)

Additional file 4: Figure S2: Parental disease resistance to the late leaf spot in the field. (TIFF 2 MB)
