# Supplementary figures and images for: Construction of a SNP-based genetic linkage map in cultivated peanut based on large scale marker development using next-generation double-digest restriction-site-associated DNA sequencing (ddRADseq)
Source: BMC Genomics. 2014 May 9;15(1):351. doi: 10.1186/1471-2164-15-351 (PMC4035077; doi:10.1186/1471-2164-15-351)

1A

2A

3A

4A

5A

6A

7A

8A

9A

10A

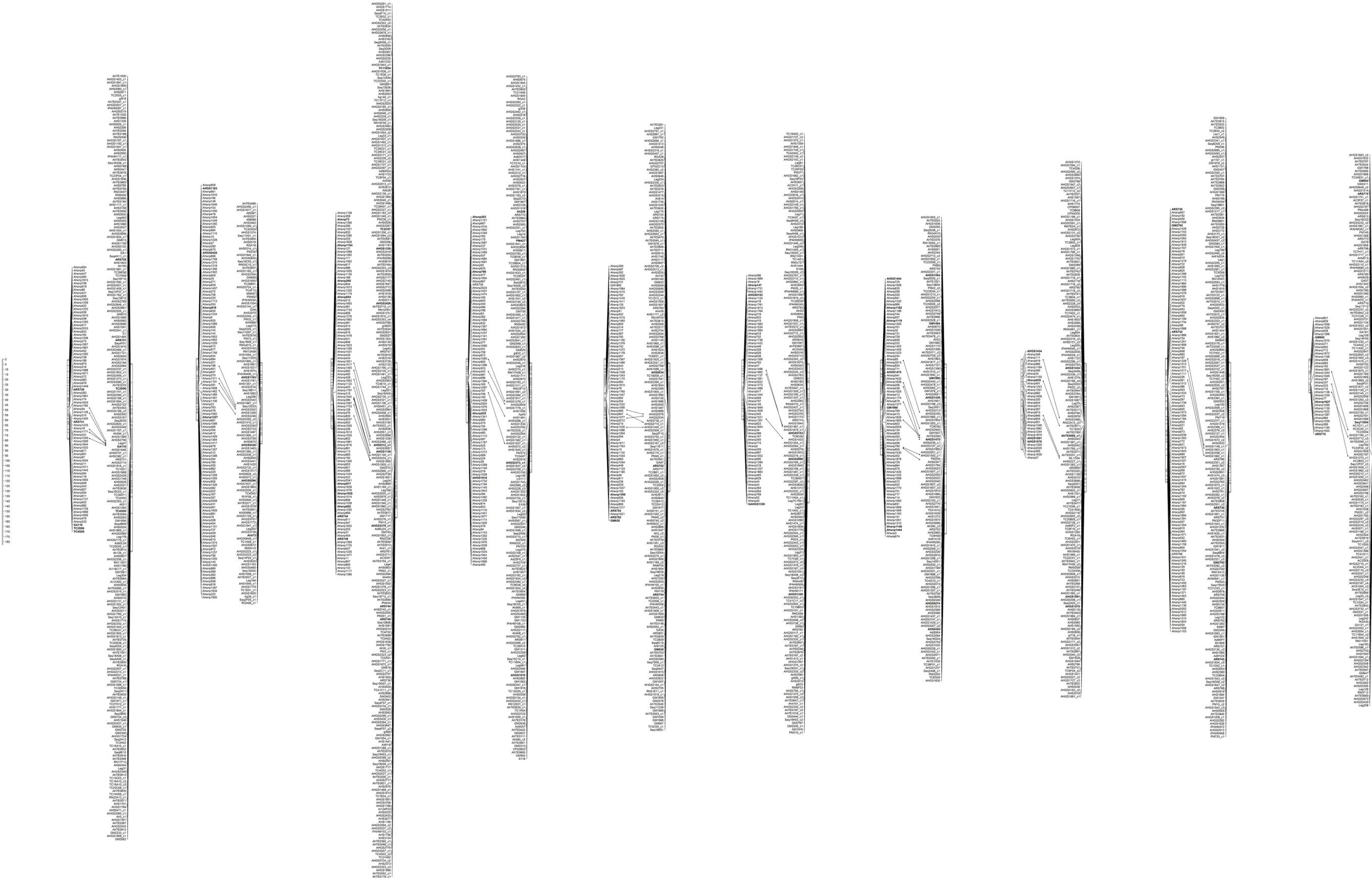

1B

2B

3B

4B

5B

6B

7B

8B

9B

10B

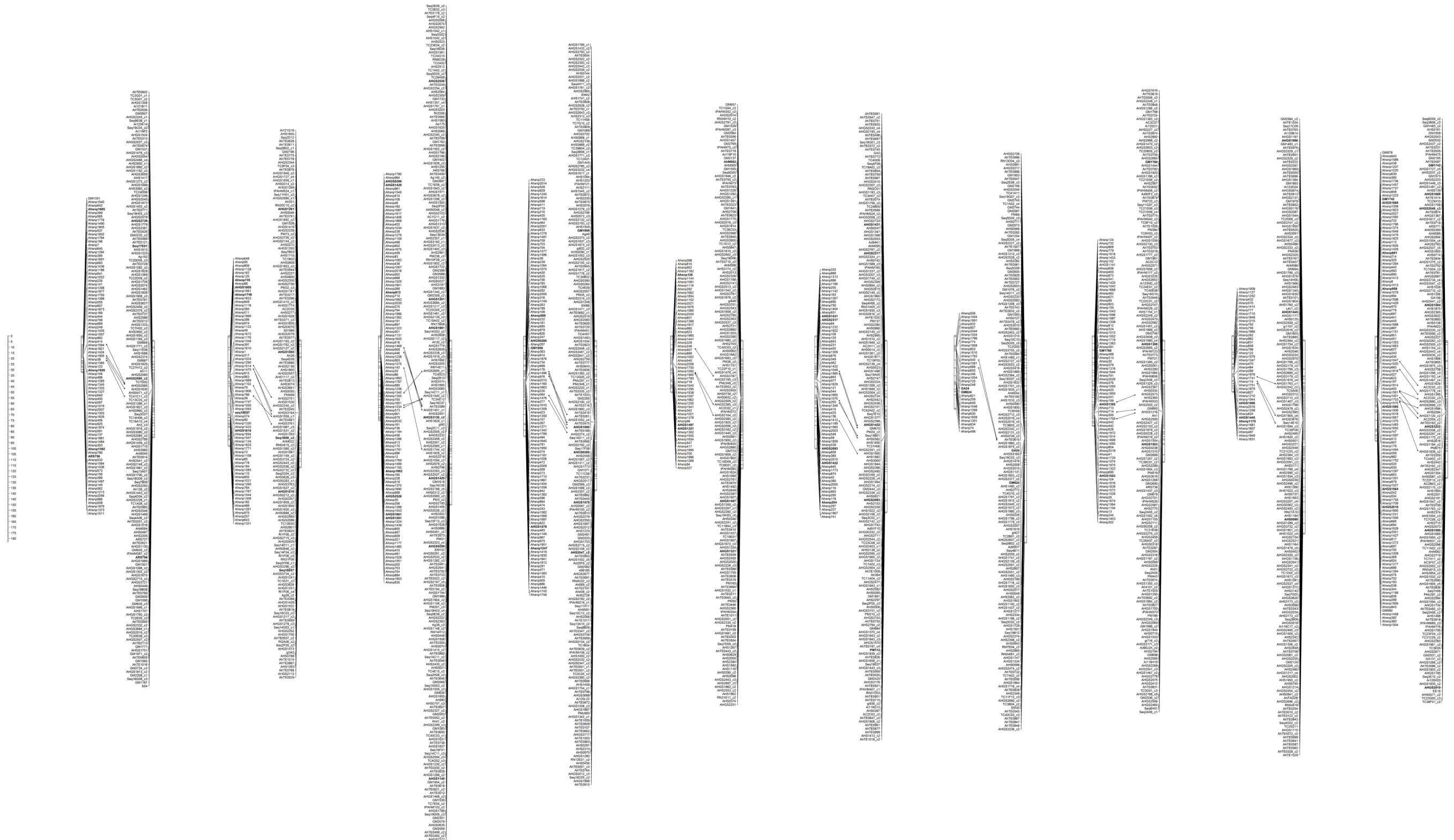

Supplement: Supplementary file 3 — Additional file 3: Figure S1: Comparison between the LGs of the SNP-based map and the integrated consensus map. For each pair of aligned LGs, the left LG corresponds to the SNP-based map, and the right LG corresponds to the integrated consensus map. Horizontal lines on the LGs indicate the positions of the mapped loci. The loci of the common SSR markers and the SNP and SSR markers that have similar map positions between the corresponding LGs of the two maps are connected by black lines. (PDF 8 MB) [file 12864_2013_6048_MOESM3_ESM.pdf]

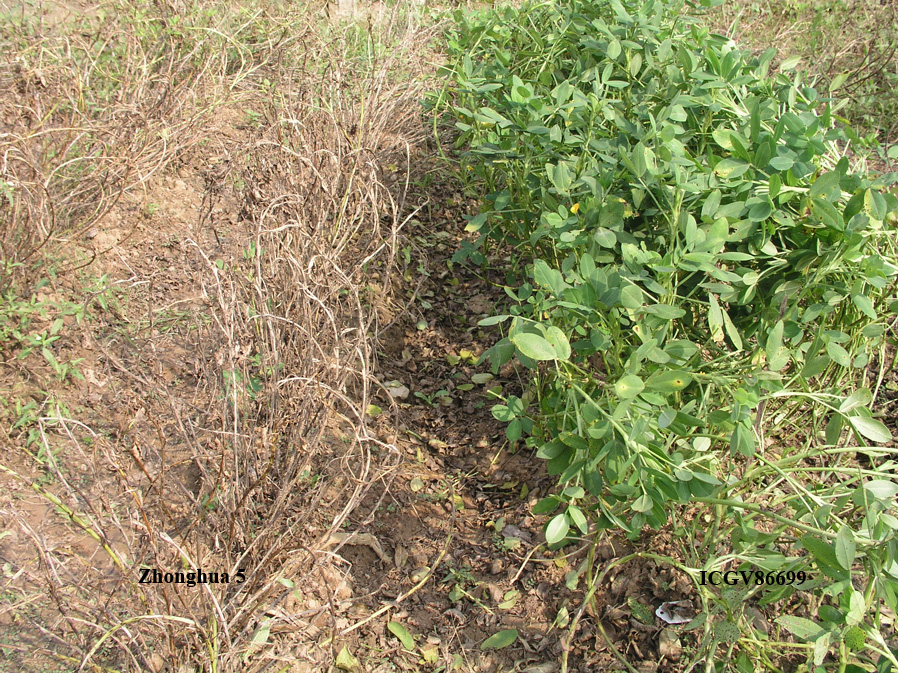

Supplement: Supplementary file 4 — Additional file 4: Figure S2: Parental disease resistance to the late leaf spot in the field. (TIFF 2 MB) [file 12864_2013_6048_MOESM4_ESM.tiff]
